# A multiobjective approach to the genetic code adaptability problem

**DOI:** 10.1186/s12859-015-0480-9

**Published:** 2015-02-19

**Authors:** Lariza Laura de Oliveira, Paulo SL de Oliveira, Renato Tinós

**Affiliations:** 1 Department of Computing and Mathematics, University of São Paulo, Ribeirão Preto, Brazil; 2 Brazilian Biosciences National Laboratory, Campinas, Brazil

**Keywords:** Genetic code, Genetic algorithm, Multiobjective approach

## Abstract

**Background:**

The organization of the canonical code has intrigued researches since it was first described. If we consider all codes mapping the 64 codes into 20 amino acids and one stop codon, there are more than 1.51×10^84^ possible genetic codes. The main question related to the organization of the genetic code is why exactly the canonical code was selected among this huge number of possible genetic codes. Many researchers argue that the organization of the canonical code is a product of natural selection and that the code’s robustness against mutations would support this hypothesis. In order to investigate the natural selection hypothesis, some researches employ optimization algorithms to identify regions of the genetic code space where best codes, according to a given evaluation function, can be found (engineering approach). The optimization process uses only one objective to evaluate the codes, generally based on the robustness for an amino acid property. Only one objective is also employed in the statistical approach for the comparison of the canonical code with random codes. We propose a multiobjective approach where two or more objectives are considered simultaneously to evaluate the genetic codes.

**Results:**

In order to test our hypothesis that the multiobjective approach is useful for the analysis of the genetic code adaptability, we implemented a multiobjective optimization algorithm where two objectives are simultaneously optimized. Using as objectives the robustness against mutation with the amino acids properties polar requirement (objective 1) and robustness with respect to hydropathy index or molecular volume (objective 2), we found solutions closer to the canonical genetic code in terms of robustness, when compared with the results using only one objective reported by other authors.

**Conclusions:**

Using more objectives, more optimal solutions are obtained and, as a consequence, more information can be used to investigate the adaptability of the genetic code. The multiobjective approach is also more natural, because more than one objective was adapted during the evolutionary process of the canonical genetic code. Our results suggest that the evaluation function employed to compare genetic codes should consider simultaneously more than one objective, in contrast to what has been done in the literature.

## Background

Amino acids differ according to side chain properties such as polarity, size and shape [1]. Proteins structural complexity and biology function variety are due to the huge number of possibilities that these building blocks can be assembled. The particular sequence order of the amino acids in the protein is dictated by the messenger RNA according to the canonical genetic code. This code maps each triplet of nucleotides, known as codon, to amino acids. The reason why the canonical code was selected over the large number of possible codes has intrigued researchers for decades [2-11].

Because some codons codify amino acids structurally correlated to them, some authors argue that the code’s organization is a result of stereochemical interactions between amino acids and codons (or anticodons) [7]. Others suggest that the organization of the code is a result of symmetry breaking processes [12,13]. However, the most popular theory is the adaptation of the genetic code from a primitive code, possibly with a small subset of precursors amino acids [14], via natural selection towards a frozen state [3], i.e., towards a local optimum if we view the adaptability process as an optimization process. The hypothesis that the genetic code has evolved is mainly supported by the robustness of the canonical code against mutations when some amino acids properties are considered [2]. Haig and Hurst [15], and some other authors after them [5,16], showed that a very small percentage of random codes are better than the canonical code in minimizing the deleterious effects of errors in the translation process.

According to the authors in [16], two approaches can be used to analyze the genetic code adaptability by natural selection. In the first one, used by Haig and Hurst [15] and known as the statistical approach, a huge set of random codes are generated. Then, the number of random codes better than the canonical genetic code is estimated using a evaluation function with only one objective, usually the robustness against mutations considering an amino acid property. However, when evaluation functions with good quality are used, it is very hard to find random codes better than the canonical code. This occurs because the space of all possible codes, called here genetic code space or, using the terminology of the optimization area, search space, is huge; there are more than 1.51×10^84^ possible codes mapping the 64 codons into 20 amino acids and three stop codons [17]. In order to identify regions in the huge genetic code space where best codes according to a given evaluation function can be found, the engineering approach was proposed. In the engineering approach, the canonical code is compared with the best codes obtained by an optimization algorithm.

An example of the engineering approach is the work of Santos and Monteagudo [18], where a Genetic Algorithm (GA) was employed to search the best codes according to an evaluation function. GAs are population metaheuristics used in optimization, i.e., instead of optimizing one solution each time, a set of solutions (population) is optimized in parallel [19]. The GA described in [18] uses an evaluation function based on only one objective to select the best solutions. The objective in this case is also the robustness against mutations considering an amino acid property. Other engineering and statistical methods also use only one objective to evaluate the genetic codes, generally also a robustness-based function [5,20].

In the paper of 1991, Haig and Hurst computed the code robustness for four amino acid properties: polar requirement, hydropathy index, molecular volume and isoeletric point. They observed that the canonical code is extremely robust for the first three properties, but it is not robust for isoeletric point. Santos and Monteagudo [18] reached similar conclusions using the engineering approach. In both papers, and also in other works found in the literature, the amino acid properties are not used simultaneously, i.e., the evaluation of the codes is monoobjective. In both works, polar requirement was considered the most relevant property to compute the robustness of the genetic codes.

Many real-world optimization problems involve conflicting objectives, having in this way a set of optimal solutions [21]. In fact, according to [22], seldom problems are monoobjective in practice. An example of multiobjective problem frequently found in industry is maximizing the product’s quality while minimizing the production’s cost. The natural selection process is also multiobjective. The natural evolution occurs in a huge search space with a large number of dynamic objectives being optimized at the same time. However, the genetic code adaptability problem has been addressed so far as a monoobjective problem [18].

In this work, we propose that the multiobjective approach for the genetic code adaptability problem is more realistic and produces more interesting results than the monoobjective approach. We propose that robustness considering polar requirement is not the only objective adapted during the evolutionary process and that other objectives should be considered simultaneously when searching for best codes and comparing them with the canonical code. Here, we test our hypothesis using a multiobjective approach where the evaluation function considers two objectives at the same time: robustness against errors considering the polar requirement [5,15,18,20] and as second objective, we test robustness against errors considering hydropathy or molecular volume. These two properties have robustness levels lower than polar requirement, but they can be still relevant.

Following the methodology presented in [18], we use a GA as optimization algorithm in order to obtain the best genetic codes and compare them with the canonical genetic code. It is important to observe that other optimization algorithms could be employed. However, since GAs use a population of solutions during optimization, they represent a direct approach to deal with more than one objective, being successfully employed in several multiobjective problems [21,23]. When compared with the monoobjective approach, more than one optimal genetic codes are obtained in the multiobjective approach. In the experimental results presented here, genetic codes closer to the canonical code are generated by the multiobjective GA.

## Methods

In many optimization problems, more than one objective should be optimized at the same time [22]. When the evaluation of only one objective should be minimized, a solution *x* is considered better than a solution *y* if *f*(*x*)<*f*(*y*), where *f*(*x*) is the evaluation of the objective that should be minimized. For example, in the statistical and engineering approaches, *x* represents a genetic code and *f*(*x*) generally is based on the robustness of the code taking in account one amino acid property.

When two objectives are considered, the comparison is more complex because a solution *x* can have a better *f*_1_(*x*) but a worse *f*_2_(*x*), where *f*_1_(*x*) and *f*_2_(*x*) are respectively the evaluations of the objectives that should be minimized. In the case where *x* has evaluations of all objectives equal or better than *y*, and at least one better, we say that solution *x* dominates *y* (Solution *y* dominates solution *x* in the opposite case). Otherwise, *x* and *y* are nondominated solutions or Pareto optimal solutions. In this way, while we are interested in only one optimum solution in monoobjective optimization, the algorithm should find a set of Pareto optimal solutions otherwise, i.e., the algorithm should find a set of nondominated solutions in multiobjective optimization [19].

There are a variety of algorithms for multiobjective optimization [24]. Among them, approaches based on GAs are very popular because the set of nondominated solutions can be represented in a natural way by the population of solutions of the algorithm. In [23], more than 4000 references of Evolutionary Computation applied to multiobjective problems are listed. Here, we employ the Nondominated Sorting Genetic Algorithm II (NSGA-II) [19], that is a state-of-art multiobjective approach when the number of objectives is not high. The NSGA-II presents good computational performance: its complexity is at most *O*(*M* *N* ^2^), where *M* is the population size and *N* is the number of objectives. Moreover, the algorithm has a mechanism for maintenance of solutions’ diversity and is elitist [19].

### Evaluation of the genetic codes

In the experiments presented in this work, the genetic codes are simultaneously evaluated based on two objectives. For each one, the robustness of the code against mutations considering a given amino acid property should be maximized, or, in a similar way, the mean squared error, which is calculated using an amino acids property, should be minimized. The mean squared error is computed here as the mean value of the difference of the amino acid property for all possible changes in the codons for a given code *C* [5,15,18,25,26], i.e.:

(1) $$ M_{s}(C) = \frac{\sum_{ij} (X(i,C)-X(j,C))^{2} }{\sum_{ij}N(i,j,C)}  $$

where *X*(*i*,*C*) is the amino acid property value for the amino acid codified by the *i*-th codon for the genetic code *C*, and *N*(*i*,*j*,*C*) is the number of possible replacements between codons *i* and *j* for the code *C*. For example, when the polar requirement is used, *X*(*i*,*C*) represents the polar requirement for the amino acid codified by the *i*-th codon for the genetic code *C*. When two objectives are minimized, two values of *M*_*s*_(*C*) are computed, one for each amino acid property, e.g., polar requirement (objective one) and hidropathy (objective two).

When *M*_*s*_ is computed, the changes in codons base positions have the same importance. However, experimental data [6] show that errors in the translational process vary according to the base position within a codon. Freeland and Hurst [5] summarized the dependence of the errors based on the base positions by:

- Mistranslation of the second base is much less frequent than mistranslation in the other two bases, whereas mistranslation of the first base is less frequent than mistranslation of the third base.
- Most mistranslations of the second base are transitional.
- Most mistranslations of first base are transitional.
- The transition bias is very small in the third base mistranslation.

In this way, Freeland and Hurst proposed that those information should be added to the evaluation function previously presented when the genetic code adaptability is investigated. For this purpose, a mistranslation weight matrix is used, as shown in Table 1. The mean squared error using the mistranslation information, i.e., incorporated with the weights given by Table 1, is also tested in this paper. The new error measure is denoted *M*_*st*_.

**Table 1:** **Weights used in the computation of** ***M***_***st***_

**Weight**	**First base**	**Second base**	**Third base**
Transitions	1	0.5	1
Transversions	0.5	0.1	1

The weights represent the importance of the position base function when computing the errors in the translation process.

Haig and Hurst [15] considered the following properties to compute the mean squared error: polar requirement, hydropathy index, molecular volume and isoeletric point. They found that the canonical code is robust for all properties, with exception for isoeletric point. Santos and Monteagudo [18] also tested those properties using the Percentage of Minimization Distance (*pmd*), which is a sort of distance measure between the canonical code and the hypothetical code (a complete description of *pmd* is given in the Section Evaluation of the results). Higher values of *pmd* means greater proximity between the evaluation value of the codes. They found the following *pmd* values: 67% for polar requirement, 53% for hydropathy property, 42% for molecular volume and 23% for isoeletric point. As a consequence, the authors used polar requirement in the subsequent experiments. It is important to highlight that in both works the results obtained are from monoobjective simulations. In the experiments presented here, we use the following amino acids properties (Table 2) to compute robustness in the multiobjective approach: polar requirement [2], hydropathy [27] and molecular volume [28]. For each experiment, NSGA-II uses two objectives each time, e.g., *M*_*s*_ for polar requirement (objective one) and *M*_*s*_ for hydropathy (objective two).

**Table 2:** **Amino acids properties**[15]

**Amino acid**	**Polar**	**Hydropathy**	**Molecular**
	**Requirement (PR)**	**Index (HI)**	**Volume (MV)**
Ala	7	1.8	31
Arg	9.1	-4.5	124
Asp	13	-3.5	54
Asn	10	-3.5	56
Cys	4.8	2.5	55
Glu	12.5	-3.5	83
Gin	8.6	-3.5	85
Gly	7.9	-0.4	3
His	8.4	-3.2	96
Ile	4.9	4.5	111
Leu	4.9	3.8	111
Lys	10.1	-3.9	119
Met	5.3	1.9	105
Phe	5	2.8	132
Pro	6.6	-1.6	32.5
Ser	7.5	-0.8	32
Thr	6.6	-0.7	61
Trp	5.2	-0.9	170
Tyr	5.4	-1.3	136
Val	5.6	4.2	84

### Genetic algorithm

Two types of encodings for the solutions (genetic codes) were tested for the GA in [18]. The first one is a nonrestrictive encoding, where the allowed genetic codes map the 61 codons into 20 amino acids (three codons are reserved for signaling the end of the transcription process). The second one is a restrictive encoding, which preserves the structure blocks of the canonical genetic code, i.e., keeps the same groups of synonymous codons found in the standard code. In this sense, the canonical code information is used in the restrictive encoding to reduce the number of possible genetic codes found in the genetic code space.

**Figure 1:**
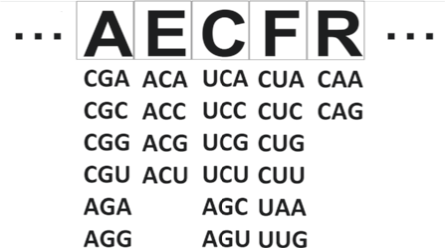
**Example of part of an individual using the restrictive encoding.** Each individual of the GA represents a hypothetical genetic code. The individual is composed by groups of codons associated to amino acids. The groups are kept as found in the canonical genetic code.

In the restrictive encoding, each individual of the GA’s population represents a code composed of 20 positions, each one related to a group of codons associated to an amino acid (Figure 1). These groups are the same found in the canonical genetic code. The stop codons are kept fixed, as in the canonical code. The restrictive encoding is used in the implementation described here.

**Figure 2:**
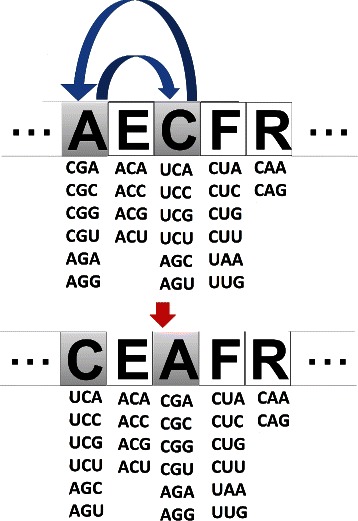
**Example of application of the swap operator.** The swap operator interchanges amino acids associated to two group of codons.

In the GA, a set of solutions (population) is allowed to evolve according to selection and transformation operators. Here, tournament selection is employed to select the individuals (solutions) to be transformed. In this operator, a percentage of individuals is randomly selected and the individual with the best evaluation is chosen. Moreover, elitism is used to preserve the best individual of each population. Like the GA described in [18], the GA employed here uses only swap as the transformation operator. As the authors in [18], we also tested a crossover operator in previous experiments, but it did not statistically improved the performance of the algorithm. In swap, amino acids associated to two groups of codons are interchanged, i.e., two positions are randomly selected and their amino acids are swapped (Figure 2).

The NSGA-II used here employs an elitist nondominated sorting to define the Pareto set [19]. The algorithm can be summarized by the following steps:

- Generate a population *P*_(0)_, which is sorted in layers according to dominance among the solutions. In this sense, the first layer corresponds to the solutions which are not dominated by other solutions. i.e., the first layer corresponds to the Pareto optimal solutions set.
- Selection and transformation operators are applied to the *P*_(*t*)_ in order to generate another population *Q*_(*t*)_. A new population *P*_(*t*)_+*Q*_(*t*)_ is then sorted according to the dominance among the solutions.
- A new population *P*_(*t*+1)_ is created, adding the initial layers of *P*_(*t*)_+*Q*_(*t*)_. When the number of individuals of the last layer exceeds the population size, a crowding distance is used to choose the most diverse individuals within a layer. The individuals are ranked according to this distance and the most diverse are added to complete the population.

The pseudo-code for the non-dominated sorting genetic algorithm II (NSGA-II) is shown in Algorithm ??.



### Evaluation of the results

In order to compare the canonical genetic code with codes in the Pareto set generated in the experiments, we use four approaches:

- Evaluation for each objective *i* for the codes in the Pareto set found by the algorithm and comparison to the evaluation of the canonical code: The values of evaluation, as well the Euclidean distance between the evaluation of the solutions in the Pareto set and the evaluation of the canonical code for each objective, are shown in tables. Also, a graphical representation for the distribution is presented. The graphical representation shows the distribution of evaluations of the nondominated solutions found by the algorithm. Each axis corresponds to one objective considered by the algorithm and the codes are represented by points.
- Percentage of Minimization Distance (*pmd*) [4] for all objectives: The *pmd* for objective *i* is computed as follows:
  (2) $$ {\fontsize{8}{12}\begin{aligned} {pmd}_{i} = 100 \times \left|\frac{\bar{f}_{i} - f_{i}(C_{canonical})}{\bar{f}_{i} - f_{i}(C)}\right| \end{aligned}}  $$
  where $\bar {f}_{i}$ is the estimated average evaluation of objective *i* for all the possible genetic codes, *f*_*i*_(*C*) is the evaluation of objective *i* for the genetic code *C*, and *C*_*canonical*_ is the canonical genetic code. The value of $\bar {f}_{i}$ is computed as the mean evaluation of objective *i* for a large number of random codes (here, 10 million codes were generated). Higher values of *p* *m* *d*_*i*_ means greater proximity between the evaluation of objective *i* for code *C* and the canonical code, relative to the estimated average evaluation for all possible codes. In order to evaluate the solutions of the Pareto set found by the algorithm, *p* *m* *d*_*i*_ is computed for all objectives minimized in the experiment.
- Dominance of the solutions in the Pareto set over the canonical code: If a code *C* in the Pareto set found by the NSGA-II dominates the genetic code, it means that the evaluation of both objectives, for the code *C*, is equal or better, and at least one is better.
- Comparison of codes of the Pareto set with the canonical genetic code: some codes of the Pareto set are shown in tables and their organization is compared with the organization of the canonical code.

## Results and discussion

In the experiments, the NSGA-II minimizes two objectives each time. The mean squared error considering the polar requirement property is always the first objective. Results of experiments with two different second objectives (mean squared error considering hydropathy index or molecular volume) are presented in this section. For each combination of objectives, experiments with *M*_*s*_ and *M*_*st*_ were generated. The values of polar requirement, hidropathy index, and molecular volume for the amino acids used here presented in Table 2.

The NSGA-II was implemented in C++ with population size equal to 100, random initial population, swap rate equal to 0.5 (each individual has a 50% change of suffering swap), and size of the tournament pool equal to 3% of the population size. For each second objective and mean squared error (*M*_*s*_ and *M*_*st*_), the NSGA-II is executed 10 times during 1000 generations with different random seeds. The results of the Pareto set obtained by combining the nondominated solutions for the 10 runs are here presented.

### Polar requirement and hydropathy index

**Figure 3:**
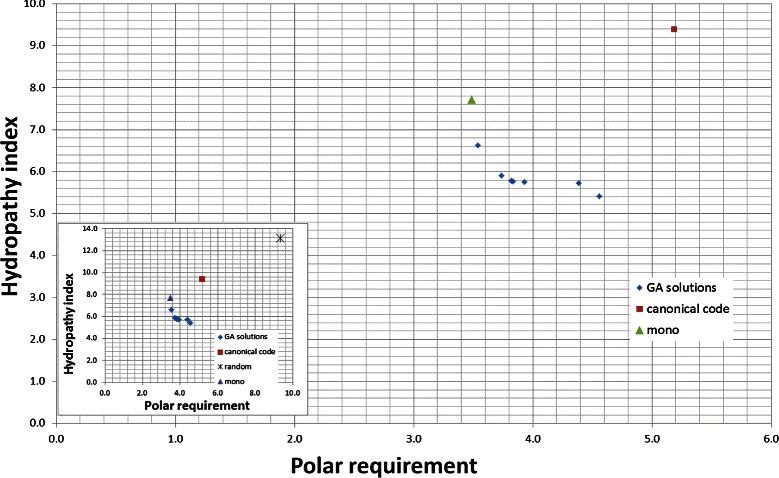
**Evaluation (** ***M***_***s***_ **) for the best codes found by the GA and for the canonical code, when polar requirement and hydropathy index are considered.** The values of *M*_*s*_ for the canonical genetic code are 5.19 for polar requirement and 9.39 for hydropathy index. The small figure includes the average evaluation value for a large number of random codes (random).

Table 3 shows the results for the nondominated codes obtained by NSGA-II using *M*_*s*_, considering the amino acids properties: polar requirement and hydropathy index. The values presented in columns 2 and 3 of Table 3 are also presented in Figure 3. The values of *p* *m* *d*_*i*_ are presented for all hypothetical codes, that dominate the canonical code. When the code does not dominate the canonical code the *p* *m* *d*_*i*_ cannot be calculated, since its values will be higher than 100*%*. The Euclidean distance from the codes to the canonical code, considering both objectives, is also presented in column 4. We use normalized values of *M*_*s*_ and *M*_*st*_ to compute the distances.

**Table 3:** **Results for the experiment with** ***M***_***s***_ **considering polar requirement and hydropathy index**

	**Obj. 1**	**Obj. 2**				
**Hypothetical code (HC)**	**PR**	**HI**	**Dist.**	**Dom.**	**pmd PR (%)**	**pmd HI (%)**
1	3.735	5.897	0.239	Yes	73.975	51.847
2	3.820	5.781	0.243	Yes	75.117	51.028
3	4.386	5.721	0.234	Yes	83.720	50.617
4	3.927	5.747	0.242	Yes	76.610	50.796
5	3.835	5.767	0.244	Yes	75.328	50.933
6	4.561	5.415	0.250	Yes	86.793	48.616
7	3.540	6.622	0.207	Yes	71.479	57.607

The values of *M*_*s*_ for the canonical genetic code are 5.19 for polar requirement and 9.39 for hydropathy index.

Figure 3 also presents the *M*_*s*_ value of an optimal code obtained by a monoobjective GA and presented in [18]. We calculated for this code, the value of *M*_*s*_ using the hydropathy index and molecular volume. It is possible to observe that the code generated by the monoobjective approach has a lower value for *M*_*s*_ with polar requirement, but a higher value of *M*_*s*_ with hydropathy index, what is expected, since only polar requirement was minimized in the optimization process.

The best *p* *m* *d*_*i*_ considering polar requirement obtained among the nondominated codes was 86.793%, while the best *p* *m* *d*_*i*_ obtained for the hydropathy index was 57.607%. It is important to highlight that, as two objectives are considered in the Pareto approach, the best *p* *m* *d*_*i*_ does not necessarily correspond to the code with the lowest evaluation for the *i*−*t* *h* objective, but to the genetic code in the nondominated set with evaluation of *i*-th objective closest to the evaluation of the same objective for the canonical code. The best *p* *m* *d*_*i*_ considering *M*_*s*_ with polar requirement obtained by the monoobjective approach in [18] was 71%. In this way, using two objectives instead of one, we obtained best codes with evaluation of the mean squared error considering polar requirement closer to the evaluation of the canonical code.

**Figure 4:**
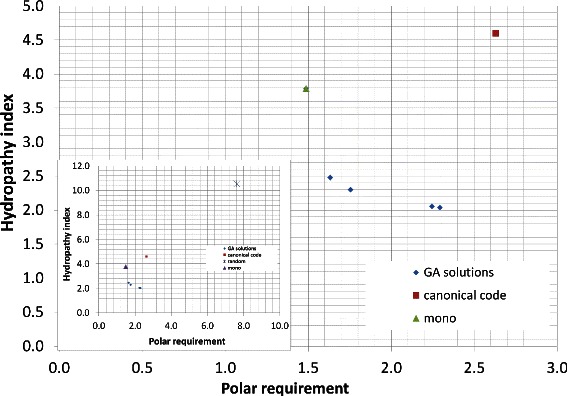
**Evaluation (** ***M***_***st***_ **) for the best codes found by the GA and for the canonical code, when polar requirement and hydropathy index are considered.** The values of *M*_*st*_ for the canonical genetic code are 2.63 for polar requirement and 4.6 for hydropathy index.

Table 4 and Figure 4 show the results for the experiment with *M*_*st*_, i.e., considering weights for mistranslation and base position errors. All the solutions found by the algorithm dominate the canonical code. Figure 4 also shows the monoobjective code obtained by [18], the monoobjective has a higher value of *M*_*st*_ with hydropathy index when compared with the codes obtained with the multiobjective approach.

**Table 4:** **Results for the experiment with** ***M***_***st***_ **considering polar requirement and hydropathy index**

	**Objective 1**	**Objective 2**				
**HC**	**PR**	**HI**	**Dist.**	**Dom.**	**pmd PR (%)**	**pmd HI (%)**
1	2.294	2.038	0.312	Yes	93.731	69.842
2	2.246	2.053	0.311	Yes	92.898	69.970
3	1.755	2.297	0.301	Yes	85.127	72.041
4	1.632	2.477	0.292	Yes	83.379	73.649

The values of *M*_*st*_ for the canonical genetic code are 2.63 for polar requirement and 4.6 for hydropathy index.

The best *p* *m* *d*_*i*_ considering polar requirement obtained among the nondominated codes was 93.731%, while 73.649% was reached for the hydropathy index. Both values are better than those obtained in the experiment with *M*_*s*_, indicating that using the weights for mistranslation errors generates a much better evaluation. Here, the best *p* *m* *d*_*i*_ for *M*_*st*_ with polar requirement was also better than that obtained in the monoobjective approach presented in [20], that was 84%.

### Polar requirement and molecular volume

**Figure 5:**
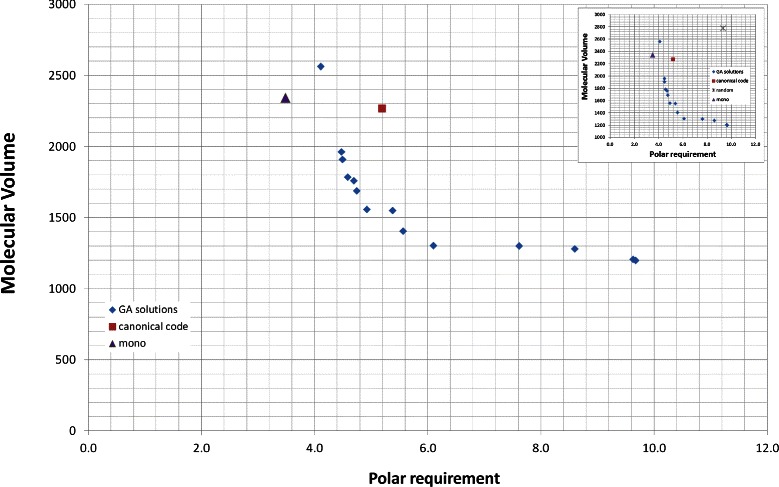
**Evaluation (** ***M***_***s***_ **) for the best codes found by the GA and for the canonical code, when polar requirement and molecular volume are considered.** The values of *M*_*s*_ for the canonical genetic code are 5.19 for polar requirement and 2266.13 for molecular volume.

**Figure 6:**
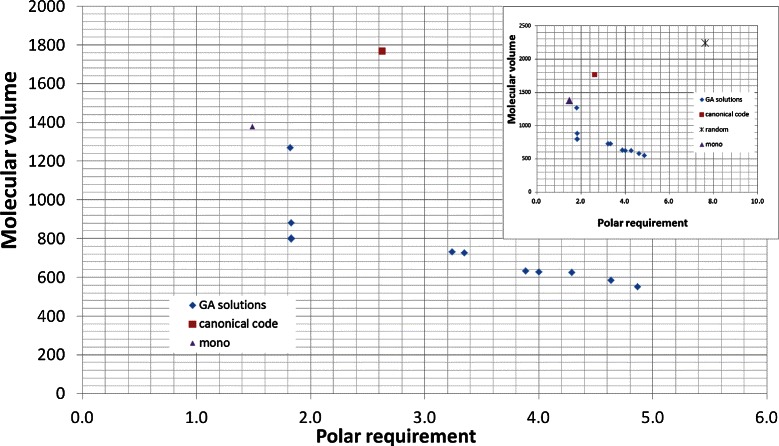
**Evaluation (** ***M***_***st***_ **) for the best codes found by the GA and for the canonical code, when polar requirement and molecular volume are considered.** The values of *M*_*st*_ for the canonical genetic code are 2.63 for polar requirement and 1766.77 for molecular volume.

Table 5 and Figure 5 show the results using *M*_*s*_ for polar requirement and molecular volume, while Table 6 and Figure 6 show the results for *M*_*st*_. The *M*_*s*_ calculated for the canonical genetic code is 5.19 and 2266.13 when molecular volume is considered. When *M*_*st*_ is calculated, the values obtained were 2.63 for polar requirement and 1766.77 for molecular volume.

**Table 5:** **Results for the experiment with** ***M***_***s***_ **considering polar requirement and molecular volume**

	**Objective 1**	**Objective 2**				
**HC**	**PR**	**MV**	**Dist.**	**Dom.**	**pmd PR (%)**	**pmd MV (%)**
1	5.380	1550	0.181	No	−	-
2	4.493	1907.460	0.103	Yes	85.587	58.945
3	9.628	1206.300	0.414	No	-	-
4	4.480	1961.500	0.092	Yes	85.345	62.832
5	9.673	1198.390	0.418	No	-	
6	4.587	1784.450	0.129	Yes	87.280	51.670.832
7	4.111	2562.610	0.107	No	-	-
8	7.617	1299.320	0.299	No	-	-
9	4.693	1759.040	0.133	Yes	89.272	34.753
10	4.745	1687.680	0.149	Yes	90.284	50.385
11	5.565	1404.140	0.219	No	89.272	50.403
12	6.099	1303.940	0.251	No	90.284	47.097
13	8.599	1278.680	0.348	No	-	-
14	4.927	1557.320	0.180	Yes	94.012	42.080

The values of *M*_*s*_ for the canonical genetic code are 5.19 for polar requirement and 2266.13 for molecular volume.

**Table 6:** **Results for the experiment with** ***M***_***st***_ **considering polar requirement and molecular volume**

	**Objective 1**	**Objective 2**				
**HC**	**PR**	**MV**	**Dist.**	**Dom.**	**pmd PR (%)**	**pmd MV (%)**
1	3.884	632,507	1134.264	No	-	-
2	1.829	802,054	964.716	Yes	86.207	33.018
3	1.829	797,364	969.406	Yes	86.214	32.911
4	1.821	1269,770	497.001	Yes	86.086	48.897
5	3.241	732,760	1034.010	No	-	-
6	3.348	726,481	1040.289	No	-	-
7	1.828	881,415	885.355	Yes	86.192	34.944
8	4.001	628,784	1137.987	No	-	-
9	4.288	624,356	1142.415	No	-	-
10	4.866	551,263	1215.509	No	-	-
11	4.631	584,045	1182.727	No	-	-

The values of *M*_*st*_ for the canonical genetic code are 2.63 for polar requirement and 1766.77 for molecular volume.

For the experiment with *M*_*s*_, 8 out of 14 solutions found by the GA do not dominate the canonical code. The best *p* *m* *d*_*i*_ found among the solutions that dominate the canonical code was 94.012% for polar requirement and 62.832% for molecular volume. For the experiment with *M*_*st*_, 7 out of 11 solutions found by the GA do not dominate the canonical code. The best values obtained considering mistranslations and base position errors were 86.214% for polar requirement and 48.897% for molecular volume. Unlike the experiment with the hydropathy index, the best results for *p* *m* *d*_*i*_ for both objectives were found in the experiment with *M*_*s*_. However, more solutions that do not dominate the canonical code were found in the experiment with *M*_*st*_.

Figures 5 and 6 also show the position of the monoobjective code obtained by [18]. In Figure 5, the monoobjective solution is far from the Pareto front and has a high value for *M*_*s*_ with molecular volume, but a low value for *M*_*s*_ with polar requirement. The monoobjective code of the Figure 6 presents the same behavior, but its position is closer to the Pareto front.

### Statistical approach

In order to compare the results obtained with the engineering approach, experiments were also performed using the statistical approach. Table 7 shows the number of random codes which are better than the canonical genetic code when 10 million random codes are generated and the objectives are individually evaluated. The results also confirm that the use of weights for the mistranslations results in a better measure to compare the codes. One can observe that it is more difficult to find random codes better than the canonical genetic code and this difficulty is higher when polar requirement is considered. For some experiments, none random code better than the canonical code was found. In order to obtain better results, much more random codes should be generated, what shows a limitation of the statistical approach. The engineering approach allows to find best codes using a smaller number of random codes, what was demonstrated in the experiments presented in previous sections.

**Table 7:** **Number of random codes better than the canonical code**

	**PR**	**HI**	**MV**
Number of codes (*M*_*s*_)	0	9	7466
Number of codes (*M*_*st*_)	0	0	20

In this experiments, 10 million random codes were generated.

**Figure 7:**
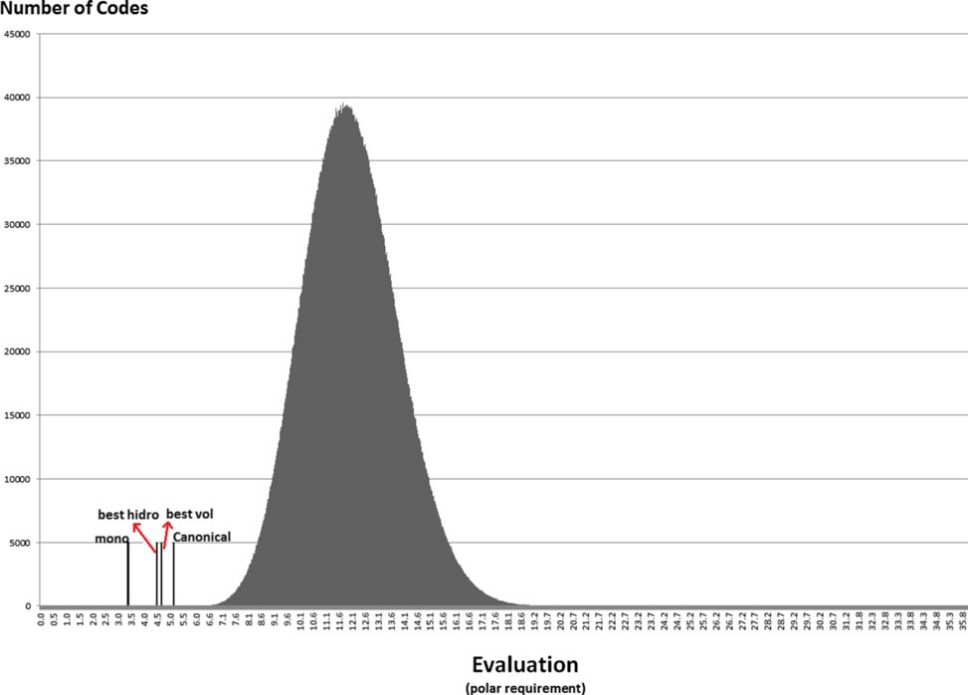
**Empirical distribution for the evaluation function, when** ***M***_***s***_ **is used with polar requirement.** For each objective, the horizontal axis shows ranges of the evaluation while the vertical axis gives the number of codes found in the respective range. The evaluation of the canonical genetic code and the objective values of the solutions with the best *pmd* obtained in the previous experiments are also plotted.

**Figure 8:**
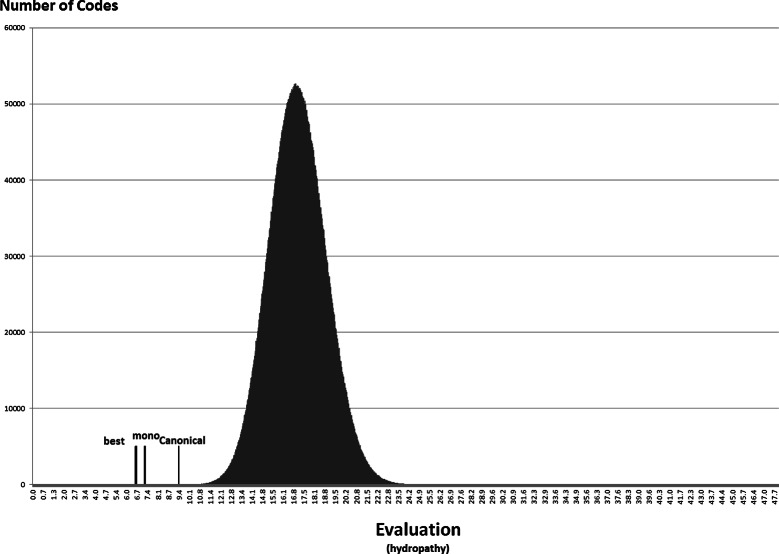
**Empirical distribution for the evaluation function, when** ***M***_***s***_ **is used with hydropathy index.**

**Figure 9:**
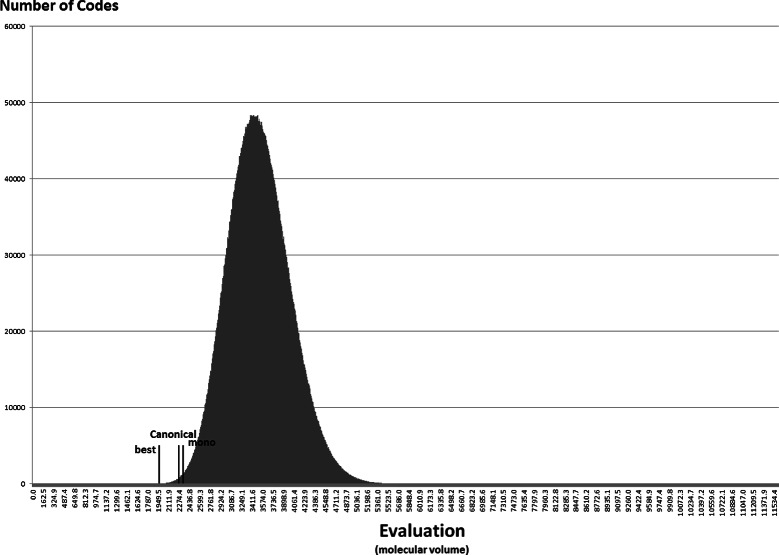
**Empirical distribution for the evaluation function, when** ***M***_***s***_ **is used with molecular volume.**

**Figure 10:**
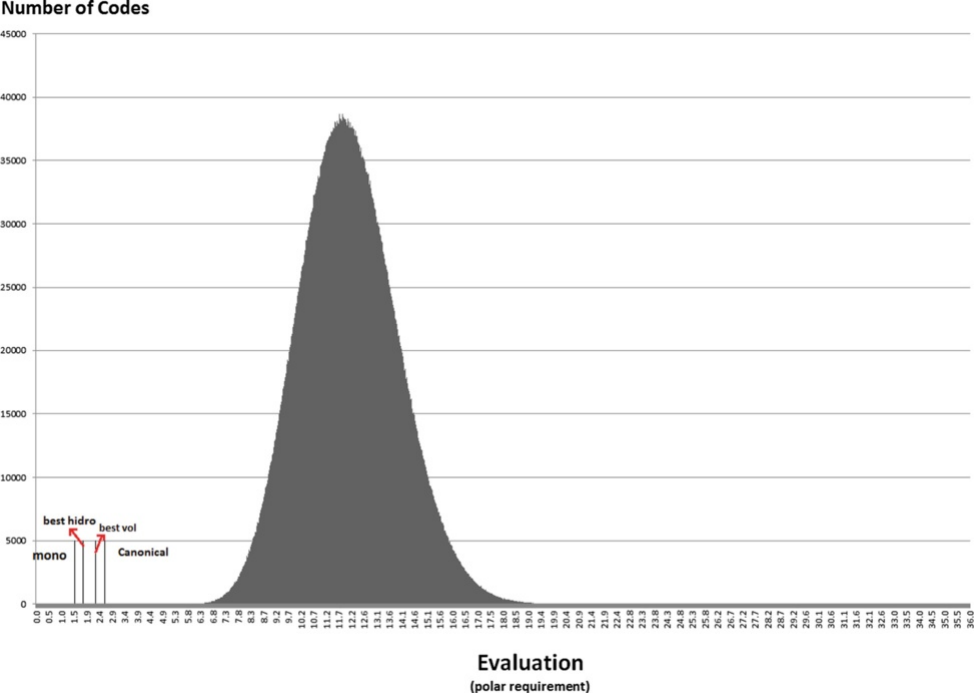
**Empirical distribution for the evaluation function, when** ***M***_***st***_ **is used with polar requirement.**

**Figure 11:**
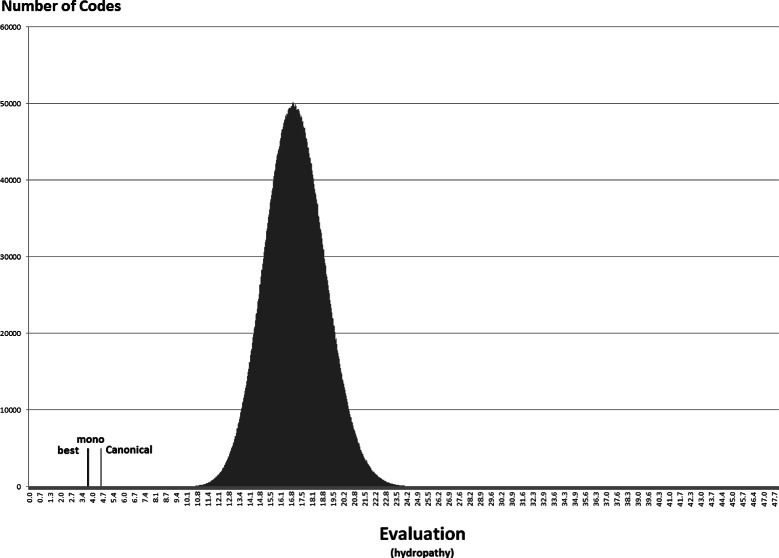
**Empirical distribution for the evaluation function, when** ***M***_***st***_ **is used with hydropathy index.**

**Figure 12:**
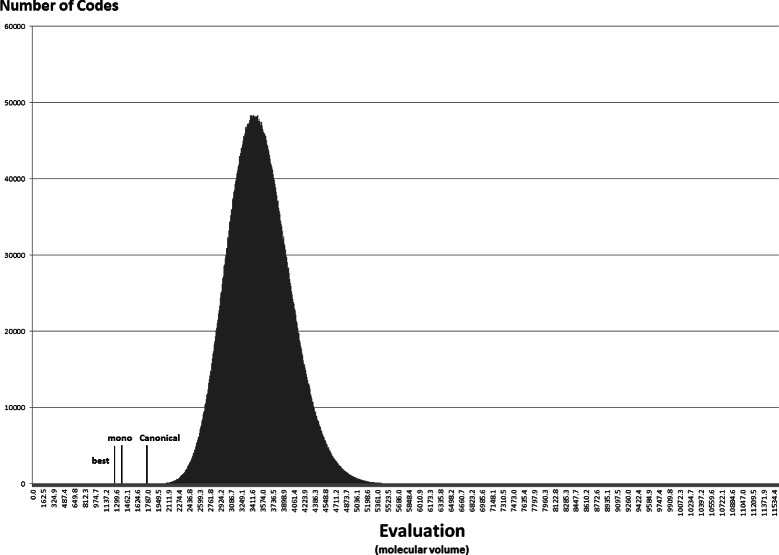
**Empirical distribution for the evaluation function, when** ***M***_***st***_ **is used with molecular volume.**

Anyway, the distribution of the random codes can be useful to show how the best codes found in the experiments with the NSGA-II compare with random hypothetical codes. Figures 7, 8, 9, 10, 11 and 12 show the empirical distribution for the 10 million random codes distributed in ranges of objective values. For each objective, the horizontal axis shows ranges of the objective value while the vertical axis gives the number of codes found in the respective range. Figures 7, 8 and 9 show the histograms of *M*_*s*_ with polar requirement, hydropathy index and molecular volume, respectively, while Figures 10, 11 and 12 show the histograms of the respective value when *M*_*st*_ is used. The objective values of the canonical genetic code and the objective values of the solutions with the best *pmd* obtained in the previous experiments are also plotted. The figures also show the position of the monoobjective code obtained by [18]. In all experiments, the evaluation of the solution found by the GA is far from the average, and smaller than the evaluation of the code with the smallest value.

In addition, in [20], the mean value for the best codes obtained using *M*_*s*_ and polar requirement was 3.506 with a standard deviation of 0.031. Here, considering polar requirement and hydropathy as objectives, the mean value of the nondominated solutions is 3.920 with a standard deviation of 0.345. When the second objective is molecular volume, the mean value of *M*_*s*_ is 5.904 with a standard deviation of 1.910. Considering that the *M*_*s*_ value of the standard genetic code is 5.19, the average value obtained in the experiments with polar requirement and hydropathy was closer than found by the monoobjective approach, i.e., it means that the hypothetical codes found by the multiobjective approach were closer to the canonical genetic code in terms of fitness values.

In the experiments presented here, the mean *M*_*st*_ for polar requirement was 2.104 with 0.569 of deviation when hydropathy is considered as the second objective and 3.233 with 1.155 of deviation when molecular volume is used as a second objective.

### Code analysis

**Figure 13:**
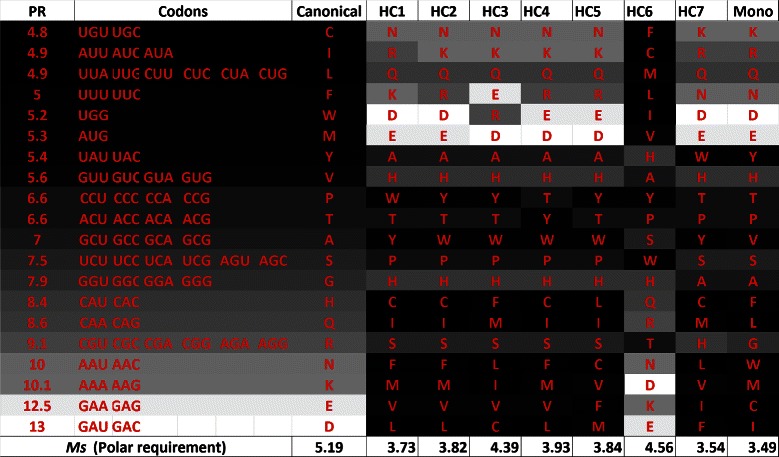
**Canonical code and hypothetical codes in a gray scale according to the respective polar requirement of the amino acids, when** ***M***_***s***_ **is used, in the experiment with polar requirement and hydropathy index.** The canonical code is shown in the first column, the polar requirement value (PR) of the amino acid in the second column, the associated codons (third column), and the amino acids for the hypothetical codes obtained in the experiment with the multiobjective GA using PR and HI are shown in the last columns.

**Figure 14:**
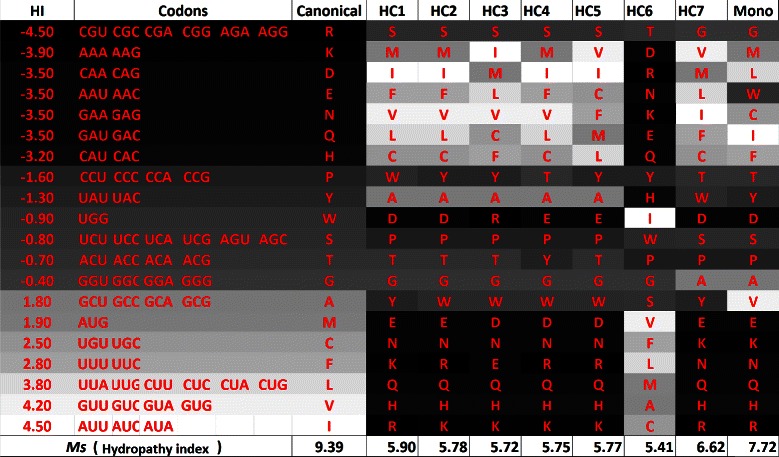
**Canonical genetic code and hypothetical codes in a gray scale according to the respective hydropathy index of the amino acids, when** ***M***_***s***_ **is used, in the experiment with polar requirement and hydropathy index.**

**Figure 15:**
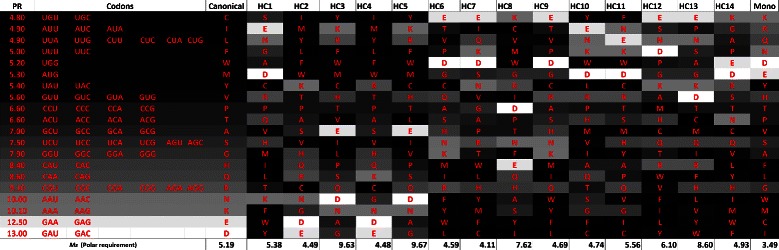
**Canonical genetic code and hypothetical codes in a gray scale according to the respective polar requirement of the amino acids, when** ***M***_***s***_ **is used, in the experiment with polar requirement and molecular volume.**

**Figure 16:**
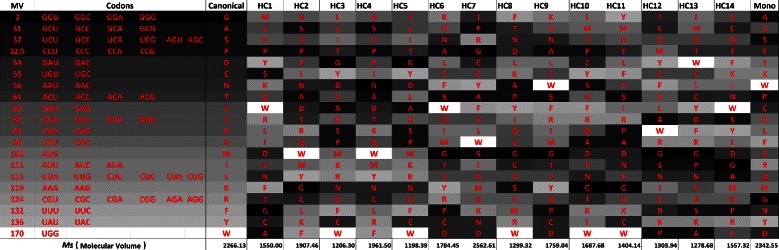
**Canonical genetic code and hypothetical codes in a gray scale according to the respective molecular volume of the amino acids, when** ***M***_***s***_ **is used, in the experiment with polar requirement and molecular volume.**

**Figure 17:**
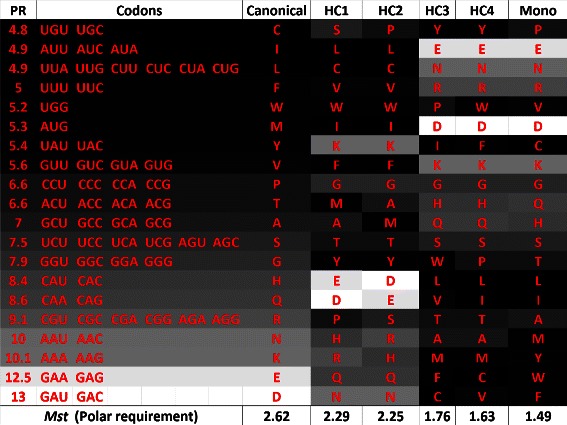
**Canonical genetic code and hypothetical codes in a gray scale according to the respective polar requirement of the amino acids, when** ***M***_***st***_ **is used, in the experiment with polar requirement and hydropathy index.**

**Figure 18:**
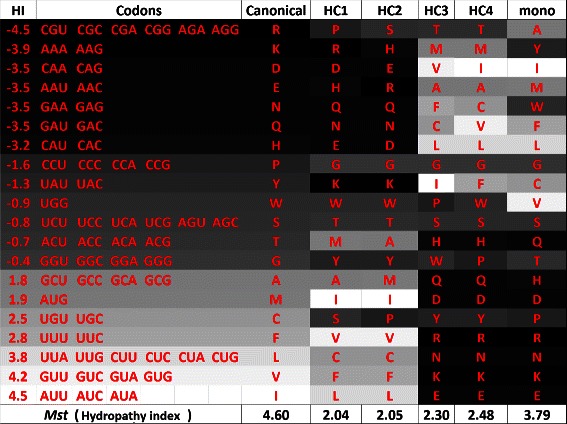
**Canonical genetic code and hypothetical codes in a gray scale according to the respective hydropathy index of the amino acids, when** ***M***_***st***_ **is used, in the experiment with polar requirement and hydropathy index.**

**Figure 19:**
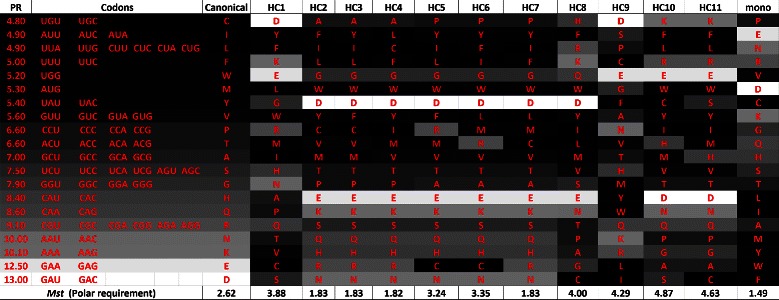
**Canonical genetic code and hypothetical codes in a gray scale according to the respective polar requirement of the amino acids, when** ***M***_***st***_ **is used, in the experiment with polar requirement and molecular volume.**

**Figure 20:**
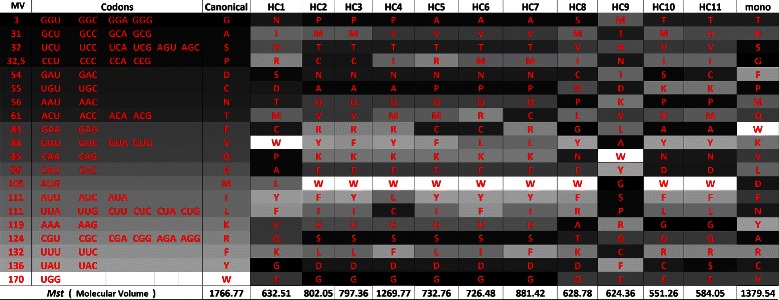
**Canonical genetic code and hypothetical codes in a gray scale according to the respective molecular volume of the amino acids, when** ***M***_***st***_ **is used, in the experiment with polar requirement and molecular volume.**

In addition, we analyze all the hypothetical codes found by the multiobjective approach. The codes were analyzed according to their values of *M*_*s*_ and *M*_*st*_. Figures 13, 14, 15, 16, 17, 18, 19 and 20 show the canonical code and the hypothetical codes colored in gray scale according to the respective amino acid properties. It is important to highlight that the codes obtained shown in Figures 13 and 14, 15 and 16, 17 and 18, and 19 and 20 are the same but they are sorted according to the respective amino acid property. In this sense, Figures 13 and 14 show the hypothetical codes obtained with polar requirement and hydropathy index using *M*_*s*_. Figure 13 shows the amino acids sorted according to polar requirement, whereas Figure 14 shows the same set of amino acids sorted according to hydropathy index. In the column “mono”, we also included the codes obtained in [18] using the monoobjective approach.

According to Figures 13 and 14, one can observe that the dark shades of the hypothetical codes are usually in the bottom of the table (except for hypothetical code 6, denoted HC6). This happens because the fitness function does not consider any relationship between the set of codons and the amino acids. In this sense, during the optimization process, it is not important to know which set of codons is linked to each amino acid. We can also observe that there is a smooth gray scale transition between the amino acids with closer values of properties. The hypothetical code 5 (HC5) is the one the the smallest Euclidean distance. HC7 is the one with the lowest value of *M*_*s*_ considering polar requirement and it is also the most similar to the code obtained using the monoobjective GA in [18], while HC6 is the one with the lowest value of *M*_*s*_ considering hydropathy index.

Figures 15 and 16 present the gray scale tables of the hypothetical codes using *M*_*s*_ considering polar requirement and molecular volume respectively. In this case the relation between the neighbors is not clear and is also difficult to observe a pattern in the figures. Despite the genetic code have a high value of robustness (*M*_*s*_) for hydropathy index and molecular volume, [29] emphasizes that the canonical code is much less optimized for molecular volume when compared to hydropathy.

Figures 17 and 18 present the gray scale analysis of the simulation with polar requirement and hydropathy index now considering *M*_*st*_, while figures 19 and 20 present the analysis considering the simulation with *M*_*st*_ and with polar requirement and molecular volume.

According to Figures 17 and 18, we can observe a smooth gradient in the gray shades. In some codes, the dark shades are on the bottom (HC3, HC4 and mono) and in others, on the top (HC1 and HC2), what is a expected behavior since no relation between the codons and the amino acids is being considered by the objectives (fitness functions). HC3 is the code with the lowest Euclidean distance, while HC4 is the one with the lowest value of *M*_*st*_ considering polar requirement and HC2 is the one with the lowest value of *M*_*st*_ considering molecular volume. HC4 is similar to the monoobjective code obtained by [18].

Observing Figures 19 and 20, we cannot see a clear pattern in the gray shades. The explanation for this behavior is again that the canonical genetic code has a poorer level of optimization when the property molecular volume is considered, as observed by [29].

Considering all the figures presented in this section, it is possible to summarize some important points:

- Low values of *M*_*s*_ or *M*_*st*_ do not necessarily imply in a structure similar to the canonical code. Usually, the hypothetical codes obtained has a small number of matches with the canonical genetic code. According to the robustness fitness function, and using the proposed optimization algorithm, it is easy to find codes more robust than the canonical code.
- It is also possible to say that the canonical code is not one of the global optimal, i.e., it is not in the Pareto front, when the multiobjective robustness-based approach is used with robustness for polar requirement as the first objective and robustness for hydropathy or molecular volume as the second objective (the same is valid for the monoobjective approach).
- We found codes similar to those found with the monoobjective in the literature, specially when the objective polar requirement is the more optimized objective. In other words, the monoobjective approach is a particular case of the multiobjective approach.
- The codes obtained with the multiobjective approach have higher values for *M*_*s*_ or *M*_*st*_ with polar requirement. This is expected, since in the multiobjective approach, more than one objective is optimized simultaneously. Similarly, the codes generated with the monoobjective approach have a higher value of *M*_*s*_ or *M*_*st*_ when considering the properties hydropathy index and molecular volume, which is also expected.
- The hypothetical codes obtained in the experiments considering hydropathy index and polar requirement have structures more similar to the canonical code and it is possible to observe a smooth gradient in the tables.
- The values of *pmd* for polar requirement are better for the multiobjective approach, when compared with the *pmd* for the monoobjective approach.

## Conclusions

In this paper, we propose a multiobjective approach to investigate the adaptability of the genetic code. Instead of using only one objective to compare the canonical code with other hypothetical genetic codes, we propose the simultaneous use of two or more objectives. In order to test our hypothesis, we investigate the multiobjective approach with two objectives based on robustness. The first objective is always the robustness for polar requirement and the second objective is the robustness for hydropathy index or molecular volume.

When compared with the monoobjective approaches described in the literature, the multiobjective approach generates better results for *pmd* considering polar requirement. In the multiobjective experiments with *M*_*st*_, the best results for *pmd* for polar requirement was 94.012% (in the experiment with hydropathy index) and 90.284% (in the experiment with molecular volume) against 84% found by the monoobjective approach presented in [18]. In other words, the hypothetical genetic codes found by the optimization algorithm have evaluation closer to the evaluation of the canonical code. The experiments with molecular volume also presented the smallest Euclidean distance to the canonical code. When molecular volume was used with polar requirement, more solutions that do not dominate the canonical code were found.

One of the most visible advantages of the multiobjective approach is to provide a set of optimal solutions to be compared to the canonical code, not just one like in the monoobjective approach currently used in the literature. The use of more than one objective seems to be a more realistic strategy and, despite of not having produced hypothetical codes identical to the canonical code, the results encourage us to search for new properties that may have been important during the evolutionary process of the canonical genetic code. One of the objectives that will be investigated in the proposed Pareto approach will be entropy [30].

Another point to be highlighted is that the genetic codes found by the multiobjective approach have a higher number of matches to the canonical code than those found by the monoobjective approach. However, the number of matches are still small. Although, as discussed in the code analysis section, the number of matches is not always a good indicative of the quality of the code and new ways to analyze hypothetical codes should be investigated in future works. Also, the multiobjective approach should be employed to investigate if the canonical code is in a local optimum in the search landscape and relations between codons and amino acids should be investigated as possible objectives.
